# Evaluation of atypical squamous cells on conventional cytology smears: An experience from a screening program practiced in limited resource settings

**DOI:** 10.4103/1742-6413.67110

**Published:** 2010-08-05

**Authors:** Bharat Rekhi, Dulhan Ajit, Santhosh K Joseph, Sonali Gawas, Kedar K Deodhar

**Affiliations:** Department of Surgical Pathology, Tata Memorial Centre, Parel, India; 1 Department of Cytopathology, Tata Memorial Centre, Parel, India

**Keywords:** Cytopathology, ASC, ASC-US, ASC-H, cervical cancer screening, conventional Pap smears

## Abstract

**Background:**

The Bethesda system (TBS) 2001 has subdivided the category of atypical squamous cells (ASC) into: ASC-US (undetermined significance) and ASC-H (cannot exclude high-grade squamous intraepithelial lesion (HSIL)). The present study is an analysis of ASC-US and ASC-H cases diagnosed in a screening program practiced in limited resource settings.

**Methods:**

During the period January 2005 to December 2008, a total of 9190 smears were received, of which 568 were unsatisfactory. Cases initially diagnosed as ASC-US (n=74) and ASC-H (n=29) on conventional cytology smears were reviewed. Biopsy and human papilloma virus (HPV) results were available in limited cases.

**Results:**

On review, diagnosis of ASC-US was retained in 49 (66.2%) of the 74 initially diagnosed ASC-US cases. Remaining 12 cases were re-labeled as negative for intraepithelial lesion or malignancy (NILM), nine as low-grade squamous intraepithelial lesion (LSIL), three as ASC-H and one case as squamous carcinoma (SCC). Similarly, on review, diagnosis of ASC-H cases was retained in 17 of the 29 initially diagnosed ASC-H cases. Seven cases were re-labeled as NILM, three as HSIL and one case each as ASC-US and SCC. Overall, 8622 cases (96.6%) were diagnosed as NILM, 72 (0.83%) as LSIL, 121 (1.40%) as HSIL, 23 (0.26%) as SCC, 50 (0.57%) as ASC-US cases, 20 (0.23%) as ASC-H, five (0.05%) as atypical glandular cells (AGC) and two cases as adenocarcinomas. Out of 50 ASC-US cases, biopsy in 23 cases showed presence of CIN 1 in 16 cases (69.5%) and CIN 2 in one case (4.34%), while the remaining six cases were negative for CIN/malignancy. The remaining 20 cases with unavailable biopsy results were HPV-positive. Out of 20 ASC-H cases, biopsy in 15 revealed CIN 2 and above in 11 cases (73.3%). Three cases (20%) revealed CIN 1.

**Conclusions:**

Critical review is helpful in further reducing the number of ASC cases. The percentage of cases with CIN 2 and above is higher with ASC-H cases. The reason for relative increase in HSILs in the present study included referral bias in the screening program.

## INTRODUCTION

Cervical cancer continues to be overall the most common cancer among females in the Indian subcontinent. In contrast to the developed countries, there has been no clinical reduction in the incidence rates of cervical cancer in the developing nations, so far. This is due to lack of effective, organized, population-based mass screening programs.[1–3] Most of the cancer screening programs in India are not organized, but intended to provide possible cancer screening benefit to the women. These programs integrate variable combinations of cytological testing with Papanicolaou (Pap) test, visual screening methods, colposcopy and guided biopsy. The major centers in India practice The Bethesda system (TBS) for reporting Pap smears on conventional cytology smears. The earlier category of atypical squamous cells (ASC) in the cervicovaginal cytology reporting was found to be a less reproducible entity, as the cytomorphological criteria for its interpretation were not well defined. Secondly, it was also frequently associated with spontaneously resolving, self-limited disease. TBS 2001 for cervicovaginal cytology has subdivided ASC into two categories i.e. atypical squamous cells of undetermined significance (ASC-US) and atypical squamous cells, cannot rule out a high-grade lesion (ASC-H). ASC-US refers to cytological changes *suggestive* of squamous intraepithelial lesion (SIL) which are qualitatively and quantitatively insufficient for a definite interpretation. These are squamous cells with increased nuclear to cytoplasmic (N/C) area; exhibit minimal nuclear hyperchromasia and irregularity in chromatin distribution or nuclear shape or nuclear abnormalities with orangeophilic cytoplasm (“atypical parakeratosis”). ASC-H refers to cytological changes that cannot exclude high-grade squamous intraepithelial lesion (HSIL). These include single cells or fragments of less than 10 cells; of metaplastic type, with larger nuclei and high N/C ratio.[4] It has been documented that 10-15% cases diagnosed with ASC-US and 30 - 40% cases with ASC-H have been found with underlying precancerous lesions i.e. cervical intraepithelial neoplasia (CIN) 2 or more, on biopsy.[5,6]

Herein, we present an analysis of cases diagnosed as ASC-US and ASC-H on conventional smears prepared at a screening program conducted by a tertiary cancer referral centre in India, along with an insight into the screening program. These cases were correlated with biopsy findings and/or with results of human papilloma virus (HPV) by hybrid capture (HC)-II testing, wherever available.

## MATERIALS AND METHODS

The present study is a retrospective study of four-year duration from January 2005 - December 2008. During this period 9190 women underwent Papanicolaou (Pap) test at a screening program in Tata Memorial Hospital, Mumbai. The Pap smears were collected by health workers who have been creating cancer awareness and motivating women across the city to attend the centre for cancer screening, including the Pap test. The cases included women presenting at the preventive oncology department for routine and annual screening.

The conventional Pap smears were collected using indigenously, “in house” prepared and sterilized swab sticks. The smears were collected from vaginal vault pool and exocervix, including squamo columnar junction, along with lower part of the endocervix. Two smears from each case were stained using modified Pap technique and were evaluated according to The Bethesda System (TBS) 2001.[4] The case findings were correlated with final histopathological diagnosis and/or human papilloma virus (HPV), oncogenic types by hybrid capture (HC) II test (Digene Gaithersberg, Qiagen USA) results, wherever available (after January 2008, since when this test was started). Biopsy and HPV results were performed on limited cases. Initially, all women were screened with Pap test and subsequently underwent both, visual inspection with acetic acid (VIA) and visual inspection with lugol’s iodine (VILI), under magnification (VIM). In case of positive results, the cases were subjected to colposcopy. On colposcopy, all cases that were suspected abnormal, including CIN1 and above, were subjected to biopsy. Out of total 9190 cases, 568 smears were unsatisfactory for evaluation and were deleted from the study. Hence the study included 8,622 smears. All the negatives were double-screened by laboratory cytotechnicians before final reporting. The cases initially diagnosed as ASC-US and ASC-H were reported by various pathologists and cytotechnicians. All cases initially diagnosed as ASC-US and ASC-H were reviewed by BR (pathologist from the gynec working group) and DA (Officer In-Charge, Senior cytotechnician). The cases diagnosed as ASC-US were recommended for HPV testing and/or follow-up after one year. However, there were limited numbers of cases available for follow-up due to poor case compliance and /or limited HPV testing. ASC-H cases were evaluated with colposcopy-guided biopsy, wherever possible. All discrepant cases, including cyto-histological discordances were reviewed as a part of quality control.

## RESULTS

**Figure 1:**
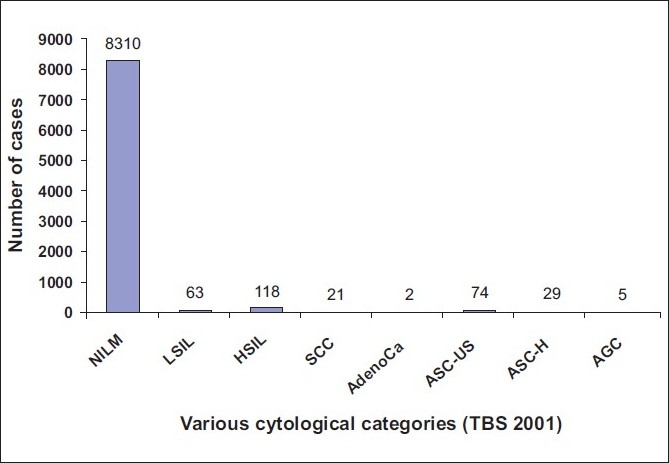
Graphical representation of original 8622 cases into various cytological categories

The age of women who underwent screening ranged from 22 - 80 years (mean = 42.6 years). Initially, out of 8622 cases, 08310 cases (96.38%) were reported as negative for intraepithelial lesion or malignancy (nilm); 181 cases (2.09%) As squamous intraepithelial lesion (sil), including 63 cases (0.73%) Of low-grade squamous intraepithelial lesion (lsil) and 118 cases (1.36%) Of high-grade squamous intraepithelial lesion (hsil); 21 cases (0.24%) As squamous carcinomas (scc) and two cases (0.02%) As adenocarcinomas. Total cases reported as asc were 103 (1.19%), Including 74 cases (0.85%) Of asc- us and 29 (0.33%) Of asc-h. Atypical glandular cells (agc) were noted in five cases [Figure 1]. ASC: SIL ratio (103 /181) was 0.56:1.

Out of 74 cases initially diagnosed as ASC-US, on review, 49 cases (66.2%) were retained with the diagnosis of ASC-US. Twelve cases were re-labeled as NILM, nine cases as LSIL, three cases as ASC-H and one case as squamous carcinoma, on review.

Out of 29 initially diagnosed ASC-H cases, 17 cases, on review, were retained with a diagnosis of ASC-H. Seven cases were ‘re-designated’ as NILM, three as HSIL and one case each as ASC-US and squamous carcinoma, respectively.

**Figure 2:**
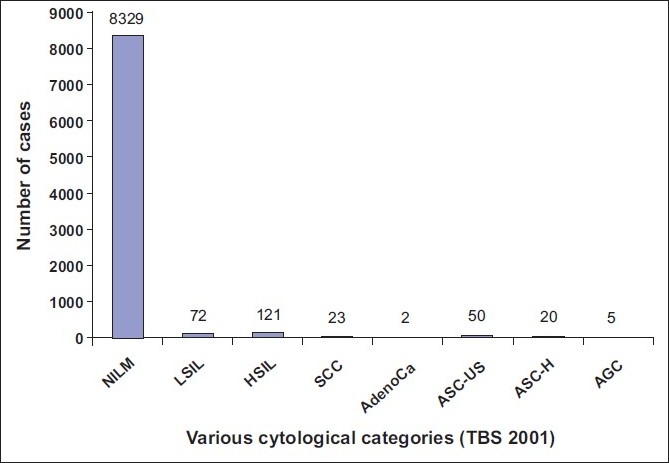
Graphical representation of 8622 cases into various categories after review of ASC-US and ASC-H cases

Finally, 8329 cases (96.6%) were diagnosed as NILM, 72 cases (0.83%) as LSIL, 121 cases (1.40%) as HSIL, 23 cases (0.26%) as SCC, 70 cases (0.81%) as ASC, including 50 cases (0.57%) as ASC-US and 20 cases (0.23%) as ASC-H, five cases (0.05%) as AGC and two cases as adenocarcinomas [Table 1] [Figure 2]. Finally, the ASC: SIL ratio (70/193) turned out as 0.36: 1. The ASC-US: LSIL ratio was 0.69: 1

**Table 1:** Comparison of variables in the present study with results from another study based on an opportunistic screening program

*Study category*	*Present study*	*Crasta et al.[15]*
Total number of cases	8622	10787
NILM	8329 (96.6)	10586 (98.14)
LSIL	72 (0.83)	20 (0.19)
HSIL	121 (1.40)	66 (0.62)
Squamous carcinoma	23 (0.26)	19 (0.18)
ASC	64 (0.74)	40 (0.38)^*^
ASC-US	50 (0.57)	40 (0.38)
ASC-H	20 (0.23)	Nil
AGC	5 (0.05)	37 (0.35)
Adenocarcinoma	2	16 (0.15)

Figures in parenthesis are in percentage

Out of 50 cases of ASC-US, biopsy results were available in 23 cases, in view of suspected abnormality on visual inspection and colposcopy. Out of these, six cases were negative for CIN, including two cases that were HPV-positive; 16 cases revealed CIN 1, including 10 cases that were HPV-positive and the remaining one case was diagnosed as CIN 2. In the remaining 27 cases, where biopsy was not performed, 20 cases were HPV-positive and seven cases were negative on two consecutive Pap tests. Percentage positivity for CIN 1 and CIN 2 with above in cases diagnosed as ASC-US was 69.5% and 4.34%, respectively.

Out of 20 ASC-H cases, biopsy was performed in 15 cases (75%). These included eight cases of CIN 3, three cases of CIN 2 (one case HPV-positive), three cases of CIN 1 and one case negative for CIN. Five cases lacked biopsy results, in view of no visible lesion on colposcopy. One of these cases was positive for HPV. Percentage of cases with CIN 2 and / or above and CIN 1, on biopsy in cases diagnosed as ASC-H, on cytology, was 73.3% and 20%, respectively. Overall, HPV results were positive in 32 (65.3%) out of 49 cases reported as ASC-US and in two (25%) out of eight cases reported as ASC-H [Table 2] [Table 3].

**Table 2:** Evaluation of ASC-US and ASC-H cases after review

*Category*	*No. of cases*	*Histology findings*					
		*Not available*	*Available*					
			*Negative*	*CIN 1*	*CIN 2*	*CIN 3*	*SCC*
asc-us	50	27 (20/27*)	6 (2/6*)	16 (10/16*)	1	-	-
Asc-h	20	5 (1/5*)	1	3	3 (1/3*)	8	-

SCC: Squamous carcinoma

(*) Indicates the number of cases with HPV positivity.

**Table 3:** ASC-US and ASC-H cases with HPV results

*Category*	*Total cases*	*HPV Performed*	*HPV positivity*	*Percentage*
ASC-US	50	49	32	65.3
ASC-H	20	8	2	25

Among 74 cases of ASC-US that were initially diagnosed, all 12 cases, on review, re-designated as NILM, were negative for CIN/malignancy, on biopsy. Out of nine LSIL cases that were ‘re-designated’ as ASC-US, five cases were HPV-positive, including one case that did not show any lesion on biopsy. In addition, two such cases (false-positives), where biopsy results were available, did not show any lesion. The reasons for over-diagnosing ASC-US in 12 ‘negative’ cases were atrophy (five cases), inflammatory reactive atypia (three cases), air-drying artifacts (three cases), ulceration and changes associated with cervical polyp (one case).

Among 29 cases of ASC-H that were reviewed, five out of the seven cases that were ‘re-designated’ as NILM, including available biopsy results, were negative for CIN/GIN/malignancy. One case ‘re-diagnosed’, as ASC-US was positive for HPV, but did not show any lesion on biopsy. Out of three cases re-labeled as HSIL, two cases, with biopsy results, revealed CIN 3, whereas one case, where biopsy was not performed in view of lack of any definite lesion on visual inspection and colposcopy, showed HPV positivity. One case re-labeled as a squamous carcinoma was confirmed on biopsy. The reasons for over-diagnosis in seven cases included immature squamous metaplasia (five cases) atrophy (one case) and inflammatory atypia (one case).

## DISCUSSION

Cervical screening programs are either of the organized or opportunistic type. In contrast to an organized screening program that includes central coordination and data collection, a defined target population with defined screening intervals; an opportunistic screening program lacks central data collection, with recruitment on self-motivation of the screening population, over variable screening intervals.[7,8] There is lack of an organized, population-based, national cancer screening program in our country. As a part of the cancer awareness and prevention program of the Tata Memorial Center, Mumbai, women get registered for early detection of cancer.

At the initial step of cervical cancer screening, Pap smear examination, followed by visual inspection and subsequently, colposcopy has been found to be useful in our settings, as documented by Shastri *et al*.,[9] wherein the authors noted sensitivity of 57.4%, 62%, 59.7%, 64.9% and 75.4% with cytology, HPV testing, VIA, visual inspection with acetic acid, under magnification (VIAM) and VILI, respectively, for detection of significant dysplasia. However, the specificity for cytology was found to be higher than visual inspection. Therefore, they recommended visual inspection, in view of high disease incidence and prevalence, along with cytology that forms the best balance of specificity and sensitivity. According to the available literature, colposcopic biopsy is often regarded as the “gold standard” on which gynecologic cytologic screening should be judged. However, some authors have demonstrated inaccuracies with histological examination.[10,11] Tritz *et al*.[11] observed that sampling and interpretation errors were the most common causes of a discrepancy between cytological and corresponding histological findings. Similarly, in a review of negative cervical biopsy specimens with corresponding cytological specimens positive for an SIL, Anderson *et al*.,[10] demonstrated 45% cases subsequently developing SIL, indicating an initial “false-negative” biopsy result, rather than “false-positive” cytological results.

Due to limited follow-up as a result of poor patient compliance, visual inspection, followed by simultaneous, Pap and colposcopic examination is a feasible screening method for increased detection of cervical neoplastic lesions in our settings. The cost factor limits HPV testing that has been identified in reducing cases with advanced cancer and cancer deaths.[12] In this study, HPV testing results were available in limited cases.

One of the significant factors for suboptimal performance of cytology-based screening in limited resource countries is poor quality of testing. The initial step of cytological evaluation is assessment of adequacy. About 6.1% cases were labeled inadequate in our study. In a multicentre organized screening trial from our country, Sankaranarayanan *et al*.,[13] observed 4.1% inadequate cases, while Gupta *et al*.,[14] in another study observed 7.1% unsatisfactory samples. Even though over 10% inadequate cases are reflective of poor quality of sampling, relatively higher rates of inadequate cases in studies from our region, in contrast to Western studies, indicate a need for improvisation of sample collection by further training of health workers, as emphasized earlier.[13] Moreover, these studies were based on conventional Pap tests. Nonetheless, the ‘inadequate’ cases were subjected to re-screening. A relative increase in HSIL (1.40%) than LSIL cases (0.83%) in the present study has also been identified in a study based upon an opportunistic screening program in southern India by Crasta *et al*.,[15] wherein the authors identified 20 cases (0.19%) of LSIL and 66 cases (0.62%) of HSIL [Table 1]. This is in contrast to other studies from the West and also in another study from our country that were based on organized population screening programs.[14] Higher numbers of HSIL cases in our study are attributable to referral bias as ours is a tertiary cancer referral centre and the screening program was not of an organized type. Moreover, the population catered, despite urban, included more women with lower socio-economic status and more cases with delayed presentations. High disease incidence could possibly be another explanation, but cannot be ascertained with this study group.

Presence of carcinoma cases, on screening, is believed to be a system ‘failure’. Such cases have been noted in earlier screening studies.[14,15] The reason is lack of an organized nation-wide, population-based screening program that could control the disease incidence. The subsequent rates could possibly be reduced if more women are targeted for Pap testing in a particular area.

One of the challenges in cervico-vaginal cytology reporting is the presence of atypical squamous cells (ASC), as was included in the earlier TBS. Later, it was observed that this category was becoming a “waste basket” for increasing percentage of lesions than were being labeled as ASC than the recommended percentage (under 5%).[16] This led to a narrower definition of ASC with a dichotomous system of qualifiers, namely ASC-US and ASC-H. While ASC-US refers to changes that are either suggestive of LSIL or SIL of indeterminate grade, ASC-H refers to changes suggestive, but not definite of HSIL.[5,6] The implications of an ASC-US diagnosis include screening at specified intervals, performing immediate colposcopy, reflex HPV testing or combination of any two, on follow-up. On the other hand, in view of high likelihood of CIN 2 and above, ASC-H warrants immediate colposcopic examination.[17] Since the inception of these two categories, there have been limited studies on these challenging lesions, especially from our region, with high cervical cancer burden.[14,15,18] Initially, our total ASC, ASC-US and ASC-H rates were 1.19%, 0.85% and 0.33%. On review, the respective rates reduced to 0.81%, 0.57% and 0.23%. The ASC: SIL ratio dropped from 0.56: 1 to 0.36: 1. The ASC-US: LSIL ratio was 0.69: 1 The lower rates were within the acceptable range and were similar to results of Crasta *et al*.,[15] wherein ASC formed 0.37% of all cases, but lower than results from Sankaranarayanan *et al*.[13] and Gupta *et al*.[14] The latter two studies were population-based screening studies. The reasons for relatively lower rates of ASC-US in our study, in contrast to the Western studies include more cases with infection, wherein inflammatory atypia becomes a close differential to ASC. The variable threshold and attempts for exact categorization, due to poor patient compliance are other possible reasons. In the present study, reduction in ASC-US and ASC-H rates, accompanied with re-categorization of cases into other definite categories, as a result of critical reviewing, reinforces the value of repeat screening of all ‘atypical’ cases, especially in limited resource settings with limited follow-up of cases. In our study, a relative higher proportion of ASC-US over ASC-H cases was in accordance with earlier studies.[15] However, the absolute number of ASC-H cases was higher.[14,19] Variable threshold levels for the diagnosis of ASC have been acknowledged earlier, where Crasta *et al*.[15] documented ASC-US cases and did not identify any ASC-H case in their series.

On review of ASC cases, all 12 cases re-designated as NILM were negative on biopsy. Whereas the reasons for over-diagnosing ASC-US lesions were mainly atrophy and inflammation, ASC-H was over-diagnosed mostly in cases of immature squamous metaplasia. The percentage of cases with CIN 1 and CIN 2 with above in cases diagnosed as ASC-US was 69.5% and 4.34%, respectively. The percentage positivity for CIN 2 with above and CIN 1 in ASC-H cases was 73.3% and 20%, respectively. This indicates a relatively higher possibility of CIN 2 and above in ASC-H cases than ASC-US cases, which have more chances of harboring CIN 1. This observation is in congruence with earlier studies and substantiates the view of immediate colposcopic assessment in ASC-H cases.[5,6,14,17] Various authors[20–22] reported different rates of high-grade dysplasia in ASC-H cases, ranging from 26 – 68% in different studies, respectively. The reasons include variable levels of threshold in interpreting ASC-H or HSIL.

Apart from not being an organized screening, the limitation in our study included lack of final outcome, especially in 27 ASC-US cases. These included 20 HPV-positive cases. The cases with HPV positivity did not have biopsy results due to lack of definite lesions. With such limitations, the practice of “see and treat” in our settings with visual examination and colposcopy seems reasonable. The limitation of opportunistic programs is lack of wide coverage.[23] Moreover, these can create a bias, as noted in our study. Nonetheless, sustained efforts, including invitations from health workers to women for more participation can add to the value of such screening programs.

## CONCLUSION

Our study describes a screening approach practiced in limited-resource settings that offers screening benefit to at least some women. A higher number of inadequate cases and referral bias for high-grade lesions is acknowledged. Sustained efforts in pursuing screening programs with a focus towards improved sample collection need to be encouraged. There is a pressing need for a population-based organized screening for wider coverage. Critical review of ASC cases with intent of ‘filtering’ cases into more definite diagnostic categories for a timely management is useful, especially in our settings where patient compliance is poor. The chances of CIN 2 and above are more with ASC-H than ASC-US cases.

## DECLARATION

This was an intradepartmental study and a substantial part of this study was presented at the CYTOCON conference (38th Annual Conference of Indian Academy of Cytologists) from 13th - 16th November 2008, Ahmedabad, Gujarat, India

## COMPETING INTEREST STATEMENT BY ALL AUTHORS

No competing interest to declare by any of the authors.

## AUTHORSHIP STATEMENT BY ALL AUTHORS

Each author acknowledges that this final version was read and approved. All authors of this article declare that we qualify for authorship as defined by ICMJE http://www.icmje.org/#author. Each author has participated sufficiently in the work and take public responsibility for appropriate portions of the content of this article.

## ETHICS STATEMENT BY ALL AUTHORS

The study was undertaken as an Intradepartmental cytology-histology correlation/audit and an academic exercise. IRB approval was not taken. At all times, patient confidentiality was maintained. Authors take responsibility to maintain documentation in this respect.

## EDITORIAL / PEER-REVIEW STATEMENT

To ensure integrity and highest quality of CytoJournal publications, the review process of this manuscript was conducted under a double blind model (authors are blinded for reviewers and reviewers are blinded for authors) through automatic online system.

## References

[1] Vallikad E (2006). Cervical cancer: The Indian perspective. Int J Obstet Gynaecol.

[2] Curado MP, Edwards BK, Shin HR (2007). In: Cancer incidence in five continents.

[3] Ferlay J, Parkin DM, Pisani P (2004). Globocan 2002: Cancer incidence, mortality and prevalence worldwide. IARC Cancer Base no 5, version 2.0.

[4] Solomon D, Davey D, Kurman R, Moriarty A, O'Connor D, Prey M (2002). The 2001 Bethesda System: terminology for reporting results of cervical cytology. JAMA.

[5] (2003). The ASCUS-LSIL Triage Study (ALTS) Group: Results of randomized trial on the management of cytology interpretations of atypical squamous cells of undetermined significance. Am J Obstet Gynecol.

[6] Sherman ME, Solomon D, Schiffmann M (2001). A comparison of equivocal LSIL and equivocal HSIL cervical cytology in ASCUS LSIL triage study. Am J Clin Pathol.

[7] Schneider V (2000). Cervical cancer screening, screening errors and reporting. Acta Cytol.

[8] Hakama M, Monsonego Franco J (1997). Screening for cervical cancer: Experience of the Nordic countries. New developments in cervical cancer screening and prevention.

[9] Shastri S, Dinshaw K, Amin G, Goswami S, Patil S, Chinoy R (2005). Concurrent evaluation of visual, cytological and HPV testing for early detection of cervical neoplasia in Mumbai, India. Bull WHO.

[10] Anderson MB, Jones BA (1997). False positive cervicovaginal cytology: a follow-up study. Acta Cytol.

[11] Tritz DM, Weeks JA, Spires SE, Sattich M, Banks H, Cibull ML (1995). Etiologies for non-correlating cervical cytologies and biopsies. Am J Clin Pathol.

[12] Sankaranarayanan R, Nene BM, Shastri SS, Jayant K, Muwonge R, Budukh AM (2009). HPV screening in rural India. N Engl J Med.

[13] Sankaranarayanan R, Thara S, Sharma A, Roy C, Shastri S, Mahé C (2004). Accuracy of conventional cytology: results from a multicentre screening study in India. J Med Screen.

[14] Gupta S, Sodhani P, Chachra KL, Singh V, Sehgal A (2007). Outcome of “Atypical squamous cells” in a cervical cytology screening program: Implications for follow up in resource limited settings. Diagn Cytopathol.

[15] Crasta JA, Chaitra V, Correa M (2009). An audit of cervicovaginal cytology in ateaching hospital: Are atypical glandular cells under-recognised on cytologicalscreening?. J Cytol.

[16] Kurman RJ, Henson DE, Hebst AL, Noller KL, Schiffman MH (1994). The 1992 National Cancer Institute worksop. Interim guidelines for management of abnormal cervical cytology. JAMA.

[17] Wright TC, Cox JT, Massad LS, Twiggs LB, Wilkinson EJ (2002). ASCCP-Sponsored Consensus Conference.2001 Consensus guidelines for the management of women with cervical cytological abnormalities. JAMA.

[18] Mulay K, Swain M, Patra S, Gowrishankar S (2009). A comparative study of cervical smears in an urban hospital in India and a population-based screening program in Mauritius. Ind J Pathol Microbiol.

[19] Sherman M, Abdul Karim FW, Berek JS, Powers CN, Sidaway MK, Tabbara SO, Solomon D, Nayar R (2004). Atypical squamous cells. The Bethesda System for reporting cervical cytology definition, criteria and explanatory note.

[20] Selvaggi SM (2003). Reporting of atypical cells, cannot exclude a high-grade squamous intraepithelial lesion (ASC-H) on cervical samples: is it significant?. Diagn Cytopathol.

[21] Ali PM, Ali SZ (2003). Atypical squamous cells of undetermined significance-rule out high-grade squamous intraepithelial lesion: cytopathologic characteristics and clinical correlates. Diagn Cytopathol.

[22] Raab SS, Bishop NS, Zaleski MS (1999). Long-term outcome and relative risk in women with atypical squamous cells of undetermined significance. Am J Clin Pathol.

[23] Norman P (1991). The potential and limitations of opportunistic screening: data from a computer stimulation of a general practice screening programme. Br J Gen Pract.

